# Amorphous calcium carbonate as a novel potential treatment for osteoarthritis in dogs: a pilot clinical study

**DOI:** 10.3389/fvets.2024.1381941

**Published:** 2024-06-25

**Authors:** Hadas Sarig-Rapaport, Sigal Krupnik, Tim G. Rowan

**Affiliations:** ^1^Amorphical Ltd., Animal Health Department, Nes Ziona, Israel; ^2^Regev Animal Hospital, Ramat Gan, Israel; ^3^Rowdix Ltd., York, United Kingdom

**Keywords:** osteoarthritis, treatment, pain, dog, mobility, disease-modifying, inflammation, drug

## Abstract

**Background:**

Amorphous calcium carbonate (ACC) is a potential new treatment for canine osteoarthritis (OA) with novel mechanisms based on local pH modulation and targeting bone remodeling, inflammation, and pain. The aim of this pilot exploratory clinical study was to obtain initial data on the potential efficacy and safety of ACC in OA dogs and to determine if further investigation was appropriate using similar assessment methods.

**Materials and methods:**

In this prospective, randomized, double-blind, controlled pilot study, 41 client-owned dogs were allocated in a 2:1 ratio to ACC: placebo given orally for 56 days. Efficacy assessments included improvements in pain and mobility using owner questionnaires [Canine Brief Pain Inventory (CBPI), Client Specific Outcome Measure (CSOM), and Veterinary Orthopedic Scores (VOS)]. Safety in the study population was monitored by veterinary examinations, clinical pathology, and adverse events.

**Results:**

Fifty-three dogs were screened, of which 41 enrolled and served for the safety assessment. Thirty-six dogs were found evaluable for initial efficacy assessment. Three dogs given placebo (21.4%) and one given ACC (4.5%) were removed before day 56 due to owner-perceived pain and were considered treatment failures. There were no serious adverse events or clinically significant treatment-related effects in the study. Overall, ACC was found safe in the small study population. On day 56, proportionally more ACC than placebo dogs were treatment successes based on CBPI (45.5% vs. 21.4%) and CSOM (63.6% vs. 30.8%, respectively); however, these differences were not statistically significant (*p* = 0.15 and 0.06, respectively). On day 56, within the ACC group but not the placebo group, the CBPI, CSOM, and VOS assessments were lower compared to day 0 and day 14 (*p* < 0.05).

**Limitations:**

The relatively small number of dogs limited the statistical power of the pilot study in evaluating the efficacy and safety of ACC.

**Conclusion:**

Study results support the conduct of larger, appropriately powered studies using similar assessments to confirm whether ACC may be a safe and effective treatment for OA in dogs.

## 1 Introduction

Osteoarthritis (OA) is a common, degenerative, chronic, and painful joint disease in dogs, characterized by joint acidity, progressive cartilage loss, and abnormal bone remodeling (1–4).

Although articular cartilage loss has been considered the main cause of OA, growing evidence suggests that the integrity and remodeling process of subchondral bone plays an important role in OA pathophysiology and clinical outcomes (5–8). Principal medications in OA include NSAIDs, which are associated with adverse reactions (4, 9), do not provide adequate pain relief in many dogs, and may not delay disease progression (10, 11). More recently, anti-nerve growth factors (NGFs) have been approved to alleviate the pain associated with osteoarthritis without a disease-modifying effect (12). Novel, effective, and safe drugs targeting bone remodeling and mineralization to preserve joint structure and relieve pain are needed.

Amorphous calcium carbonate (ACC) is a naturally occurring, non-crystalline, metastable form of calcium carbonate. It is used by crayfish to store large quantities of calcium in transient storage sites and to rapidly reabsorb calcium through the intestinal epithelia, enabling remarkably rapid remineralization of the remodeled exoskeleton (13–16). Amorphous solid dispersions, especially those based on nanosized particles, are an established method of increasing permeability, bioavailability, and biological activity, particularly of poorly soluble compounds (17, 18). A commercial, synthetically produced, stabilized, amorphous, nanosized ACC is currently used as a dietary supplement in humans and dogs.^1^ Due to its amorphous molecular structure and nanometric dimensions (primary particles with an average size of 40–60 nm), ACC has increased solubility and bioavailability. It is ~120 times more soluble and has double the oral bioavailability in humans than the conventional (crystalline) calcium carbonates (13, 14, 16). ACC’s dissolution is pH-dependent. It becomes soluble in pH below 7.35, and at pH ≥ 7.4, ACC’s solubility becomes negligible. Under physiological conditions, the pH of cartilage is weakly acidic (pH 6.9–7.2), but in OA patients, the environment can become massively acidified by the production of pro-inflammatory factors and enhanced anaerobic glycolysis (pH can drop up to 5.5), which significantly affects cartilage metabolism and inhibits matrix synthesis (19). Following its systemic absorption, ACC is postulated but not yet established to exert its effect on osteoarthritis by counteracting the local acidity in the OA joint to attenuate the consequent inflammatory joint destruction and impaired bone remodeling. Despite the stringent control mechanisms of both extra and intracellular pH essential for maintaining cellular biochemical reactions and tissue homeostasis, local pH fluctuations occur frequently under pathological conditions, and acidic extracellular microenvironments are known to characterize many neoplastic, ischemic, and inflammatory diseases, including osteoarthritis (8, 20–22). Upon its dissolution into calcium and carbonate, ACC’s basic carbonate anions can be spontaneously converted to bicarbonate (the body’s main pH buffering regulator) ions by binding protons to normalize the pH locally and modulate the inflammatory processes triggered by local acidity (22, 23). In a rat rheumatoid arthritis model, for example, levels of cathepsin K, a key cysteine acidity-activated protease involved in OA pathogenesis, pain sensation, and bone and cartilage degradation were reduced by ACC.^2^ In animal models of osteoporosis, bone resorption is remarkably reduced, while bone formation rate and mechanical bone strength are increased by oral ACC (15, 16). ACC’s effect on bone remodeling processes in inflammatory bone disorders thus advocates its research in osteoarthritis.

There are deficits in current knowledge of ACC effectiveness, speed of onset, and safety in treating OA in dogs. However, safety has been established for human use as a nutritional supplement. In this study, two previously described owner questionnaires, the Canine Brief Pain Inventory (CBPI) and Client Specific Outcome Measure (CSOM), were used as efficacy outcome measures, in addition to veterinary assessments (24–29). The utility of CBPI and CSOM in OA dogs given ACC has not previously been studied, and evidence of ACC efficacy in OA dogs is necessary to progress the development of ACC as a new animal drug.

The principal hypothesis of this study was that ACC given for 8 weeks is effective in reducing pain and improving mobility in dogs with naturally occurring OA. The objectives of this pilot exploratory study were to evaluate the potential efficacy of ACC to control pain and improve mobility in dogs with OA, based primarily on owner assessments, to determine safety in the study population, and to estimate data variability for use in the design of future definitive or confirmatory studies.

## 2 Materials and methods

### 2.1 Study design

This pilot study was designed as a prospective, randomized, double-blind, placebo-controlled clinical study. As this was the first clinical study using ACC, a larger sample size was used for ACC to increase the power to detect adverse effects. Enrolled dogs were randomly assigned to either ACC or placebo treatments in a 2:1 ratio by order of entry into the study at the participating clinics. Dogs were treated for 56 days from day 0. Study visits were conducted at baseline (day 0) and on days 14, 28, and 56. Randomization used a unique computed randomization table in blocks of three.

The study aims were to evaluate treatment success after 56 days of ACC treatment compared with placebo for standard criteria used in CBPI [decreases in both Pain Severity Score (PSS) and Pain Interference Score (PIS)] and to estimate treatment effect sizes. Additional objectives were to (i) assess treatment success rates using CSOM, (ii) observe changes in PSS, PIS, and CSOM scores and Veterinary Orthopedic Score (VOS) over time, and (iii) evaluate safety while recognizing that the small study population limits the wider applicability of safety observations.

### 2.2 Animals and eligibility

The pilot study was approved by the Animal Testing Council of Israel on 20 June 2021 (approval number 20062021). Signed owner consent for each dog was obtained before study entry. Client-owned dogs aged ≥2 years, with body weight ≥10 kg, of any breed or sex were enrolled in the study from 11 veterinary clinics in Israel. Study population demographics are shown in Table 1. Enrolled dogs must have had owner-reported signs of OA and confirmed radiologic and clinical signs of OA, including a lameness score of ≥1 using VOS. Each dog had to be in good general health as assessed by physical examination, medical history, and clinical pathology (hematology and clinical chemistry), including normal serum calcium and phosphorus concentrations. Dogs that were pregnant, lactating, or intended for breeding, or with renal or hepatic disease, neurological abnormalities, joint infections, or lameness associated with other orthopedic or neoplastic diseases that may interfere with treatment assessment were excluded. The severity of pain and its effect on the performance of daily activities were assessed by owners on day 0 (baseline, first day of treatment) using the CBPI to produce scores for PSS, PIS, and quality of life (QOL). To be enrolled, each dog had to have a mean PSS ≥2 and a mean PIS ≥3. Analgesic or anti-inflammatory medications were forbidden throughout the study, and NSAIDs, short-acting corticosteroids, long-acting corticosteroids, joint laser treatment intra-articular injections, and hydrotherapy were withdrawn 10, 14, 28, 90, and 10 days, respectively, before day 0. Dogs receiving nutraceuticals for ≥30 days before day 0 remained on that treatment. No conditions were imposed on feed or exercise given to the dogs. Dogs requiring pain medications were rescued from the study and considered treatment failures.

**Table 1:** Population demographics at enrolment of the intention to treat (ITT) and per protocol (PP) populations.

	ITT		PP	
Characteristic	Amorphous calcium carbonate (*N* = 26)	Placebo (*N* = 15)	Amorphous calcium carbonate (*N* = 22)	Placebo (*N* = 14)
**Age (years)**				
Mean (±SD)	9.9 (±3.4)	8.3 (±3.2)	9.4 (±3.4)	8.1 (±3.1)
Median	10.9	8.6	10.5	8.4
Min, Max	3.7, 13.9	2.5, 12.9	3.7, 13.9	2.5, 12.9
**Sex**				
Female intact *N* (%)	1 (3.8)	0 (0)	1 (4.5)	0 (0)
Female spayed *N* (%)	17 (65.4)	9 (60)	14 (63.6)	8 (57.1)
Male intact *N* (%)	0 (0)	0 (0)	7 (0)	0 (0)
Male castrated *N* (%)	8 (30.8)	6 (40)	7 (31.8)	6 (42.9)
**Weight (kg)**				
Mean (±SD)	27.4 (±9.7)	33.3 (±6.7)	28.1 (±10.2)	33.7 (±6.8)
Median	28.4	32.2	29.5	33.3
Min, max	10.2, 43.6	19.2, 44.7	10.2, 43.6	19.2, 44.7
**Total calcium (mg/dL)**				
Mean (±SD)	10.2 (±0.7)	10.2 (±0.7)	10.1(±0.7)	10.2 (±0.7)
Median	10.4	10.3	10.2	10.4
Min, max	8.7, 11.4	9.1, 11.6	8.7, 11.4	9.1, 11.6
**Total phosphorus (mg/dL)**				
Mean (±SD)	4.0 (±0.7)	4.0 (±0.8)	3.9 (±0.6)	3.9 (±0.8)
Median	4.1	4.1	4.0	4.1
Min, max	2.4, 5.6	2.4, 5.4	2.4, 5.0	2.4, 5.4
**Duration of clinical signs as assessed by owners (years)**				
Mean (±SD)	2.7 (±2.2)	2.7 (±3.3)	2.5 (±1.7)	2.8 (±3.4)
Median	2.0	1.2	2	1.1
Min, max	0.5, 10.4	0.0, 11.4	0.5, 8.4	0.0, 11.4
**Dogs age ≥ 10 years**				
*N* (%) ≥10 years	16 (61.5%)	5 (33.3%)	12 (54.5%)	4 (28.6%)
**Breed**				
Mixed *N* (%)	14 (53.8%)	11 (73.3%)	10 (45.5%)	10 (71.4%)
Purebred *N* (%)	12 (46.2%)	4 (26.7%)	12 (54.5%)	4 (28.6%)

### 2.3 Study procedures and treatments

At baseline, owners’ consent was obtained, followed by screening procedures to determine eligibility for enrolment. During each visit on days 0, 14, 28, and 56, owners completed CBPI and CSOM questionnaires, and veterinarians conducted orthopedic examinations and completed a VOS questionnaire. At baseline, radiographs of the joint with the severest clinical signs of OA, selected by the veterinarian as the “Study Joint,” were conducted. On days 0 and 56, veterinarians completed a full physical examination, and blood was collected for hematology and biochemistry. Dogs were treated with ACC for 56 days at a dosage of approximately 60 mg/kg (~20 mg/kg of elemental calcium) twice daily, dispensed in sequentially numbered powder-containing sachets. This dosage regimen was selected based on prior data for efficacy and safety from pre-clinical and human clinical studies. The placebo was microcrystalline cellulose powder matched to ACC by color, shape, particle size, and packaging. Owners were instructed to mix the powder with small quantities of wet food and verify the full dose was consumed. Dosing compliance was evaluated by counting returned empty sachets and owners’ diary records of administration. The product labeling and treatment coding were performed by staff in the manufacturing facility who were otherwise not involved in the study and were masked to treatment assignments in the clinics. Care was taken to ensure that owners, veterinarians, and clinic staff were masked to treatment group allocation throughout the study.

### 2.4 Outcome measures

CBPI and CSOM scores were used as efficacy endpoints for ACC treatment. The VOS questionnaire was used for veterinary assessments. Owners and veterinarians did not have access to the scores of previous assessments. The owner-completed CBPI, a two-part validated instrument assessing changes in pain severity and pain interference items [each on an 11-point (0–10) numerical scale], served as inclusion criteria and was used as described previously to assess treatment success (24–26). The owner-completed CSOM evaluated OA-related mobility impairment based on three individually selected impaired activities (e.g., climbing stairs, rising from rest, and jumping into the car) compared with when the dog was considered normal and rated by owners using a 5-point scale (1–5) to produce the total CSOM scores (sum of the three activities, range 3–15) as previously used (27). The VOS questionnaire was completed for the joint with the severest clinical signs of OA (“study joint”) and evaluated lameness at a walk (0–4) and a trot (0–4), pain on palpation or manipulation of the joint (0–3), range of motion (0–3), and joint swelling (0–3). The total VOS was the sum of scores for each of the five components (range 0–17) and was very similar to other veterinary questionnaires, such as the Total Orthopedic Score (TOS) (28). Any dog that necessitated pain medication was rescued and exited from the study, and the percentage of dogs necessitating rescue treatment was recorded for each group.

Any adverse event (AE), defined as a clinical sign considered undesirable regardless of whether or not considered treatment-related, was recorded. A serious AE (SAE) was an AE that was life-threatening and resulted in hospitalization, persistent or substantial disability, or death. Safety of ACC was evaluated by veterinary physical examinations, clinical pathology, owner observations, and AEs.

### 2.5 Sample size

This study was planned as a pilot study to obtain initial indications of potential efficacy and safety data. As this was the first clinical study using ACC in OA dogs, no preliminary values for either variance or effect size of ACC on pain and/or mobility were available for sample size calculation. To determine indicative sample size for the assessment of potential differences in efficacy between treatments, we used a previously described approach for sample size estimation of an initial or pilot clinical study, which aims to minimize the overall study sample size of a subsequent main study, adequately powered to evaluate statistical differences (30). For a main (confirmatory) study design of 90% power and two-sided 5% significance, the sample size of each treatment arm in an initial pilot study should be 75, 25, 15, or 10 for standardized effect sizes of extra small (<0.1), small (0.2, range 0.1 to <0.3), medium (0.5, range ≥0.3 to 0.7), or large (≥0.7), respectively. We anticipated that ACC would have clinically beneficial medium effect sizes on pain severity (PSS) and pain interference (PIS) determined at day 56 compared with day 0. Given a common 10–20% attrition rate observed in other canine OA studies, this initial study aimed to enroll a total of approximately 40 dogs.

### 2.6 Statistical methods

Each dog served as an experimental unit. Statistical significance was evaluated at a two-sided α = 0.05. The safety analysis was performed on the intent-to-treat (ITT) population of all dogs who were randomized and given at least one treatment dose. The evaluation of potential efficacy was conducted on the per-protocol population (PPP), a subset of the ITT group without substantial protocol violations.

The CBPI score on day 56 compared with day 0 was the *a priori* main indicator of potential efficacy and was compared between treatment groups. A predefined criterion of success was used to classify each dog as either treatment success or failure. A dog with PSS decreased by ≥1, PIS decreased by ≥2, and the same or better QOL was considered treatment success, as described previously (25, 26). Any dog not defined as a success was considered a treatment failure. An additional analysis used less conservative criteria for success defined as PSS decreased by ≥1, PIS decreased by ≥1, and same or better QOL on day 56 compared to day 0. Success defined as decreases in PSS ≥ 1 combined with PIS ≥ 2 has been widely used; however, the lesser criteria of decreases in PSS ≥ 1 and PIS ≥ 1 have also been considered on the basis that if owners can notice a difference in their pet’s level of pain, as reflected by an improvement of 1 in scores for CPBI, then that improvement is clinically relevant, although it may not be a large difference or indicate that the dog is completely pain-free (26). The proportion (%) of dogs classified as treatment success were summarized and compared between treatment groups for days 14, 28, and 56 using the N−1 Chi-squared test (31, 32).^3^ The proportion (%) of dogs rescued before day 56 was also compared between treatments using the N-1 Chi-squared test.

The CSOM scores on days 0 and 56 were compared between treatments, similarly to CBPI, to assess potential efficacy using this tool. A dog with a CSOM score decreased by ≥2 on day 56 compared to day 0 was considered a success, as described previously (27). The proportion (%) of dogs rescued before day 56 was also compared between treatments using the N-1 Chi-squared test.

The PSS, PIS, CSOM, and VOS data for days 0, 14, 28, and 56 were evaluated to explore differences between treatment groups and time to effect, using repeated measures analysis of variance and, as recommended by EMA, the baseline (day 0) measure of the response was included as a covariate (33, 34).^4^ Comparisons between means were based on *a priori* preplanned pair-wise comparisons. Within-treatment group comparisons were considered secondary to between-treatment group comparisons.

Standardized effect sizes of comparisons at day 56 between-treatment groups and within-treatment groups for PSS, PIS, and CSOM scores were calculated using Cohen’s d formula (35, 36). Odds ratios were used for assessing the effect size of treatment success (% of responders) based on CBPI and CSOM.

The last observation carried forward method was performed for dogs excluded at specific time points due to OA-related pain only. Serum biochemistry, calcium and phosphorus concentrations, hematology, physical examination data, and AEs were summarized and evaluated by the treatment group using descriptive statistics. There was no missing data except for CSOM scores for one dog in the placebo treatment group; this animal was excluded from the data analysis for CSOM only.

## 3 Results

### 3.1 Study population

**Figure 1:**
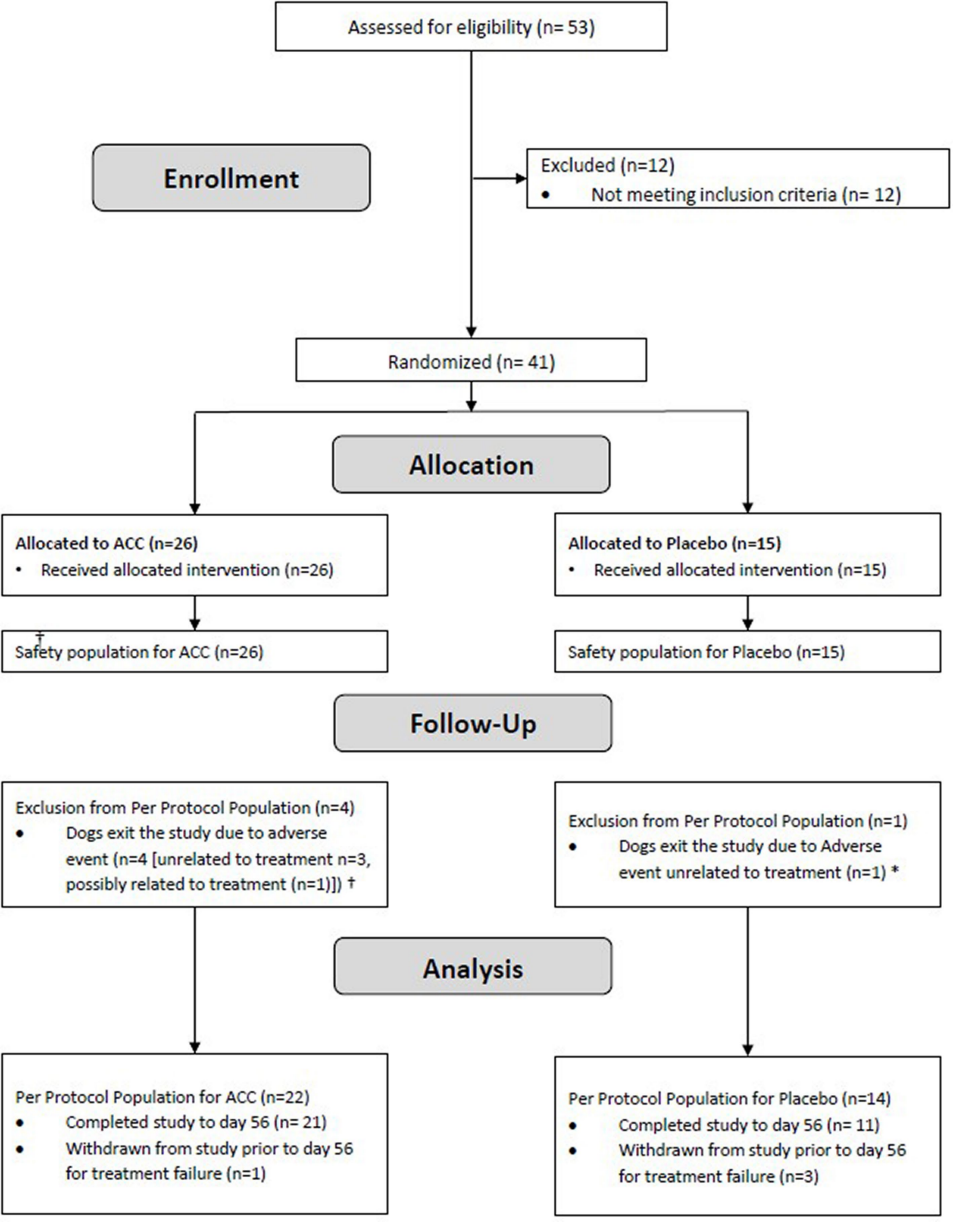
Flow diagram of study subjects. †ACC group: Unrelated to treatment (Traumatic tongue rupture, Cushing syndrome, disc protrusion), Possibly related to treatment (urinary tract infection). * Placebo group: generalized lymphadenopathy.

Enrolment began in October 2021 and was completed in March 2022. Fifty-three dogs were screened, of which 41 were enrolled and included in the safety (ITT) population and 36 dogs were included in the PPP (ACC, *n* = 22, placebo, *n* = 14; Figure 1). In the safety population, dogs ranged in age from 2.5 years to 13.9 years. Body weights ranged from 10.2 kg to 44.7 kg (Table 1). There were more females than males in the population (27 females and 14 males), but their proportions within ACC and placebo groups were similar (69.2 and 60.0%, respectively). The ACC group had a greater proportion of older dogs (≥10 years) than placebo (61.5 and 33.3%, respectively). Serum total calcium and phosphorus concentrations were similar between treatment groups.

Similarly, in the PP population, there were more females than males (23 females, 13 males), but their proportions within the ACC and placebo groups were similar (68.1 and 57.1%, respectively). The ACC group had a greater proportion (~twice) of older dogs (≥10 years) than placebo (54.5% vs. 28.6%, respectively), as well as a greater proportion (~twice) of purebred dogs (54.5% vs. 28.6%, respectively). The most common joints selected as “study joints” in both ACC and placebo groups were hips with similar proportions (68.2 and 57.1%, respectively).

### 3.2 Outcomes

Three dogs (21.4%) given placebo and one dog (4.5%) given ACC were removed from the study before day 56 for rescue treatment due to owner-perceived pain and were considered treatment failures (*p* = 0.12).

**Figure 2:**
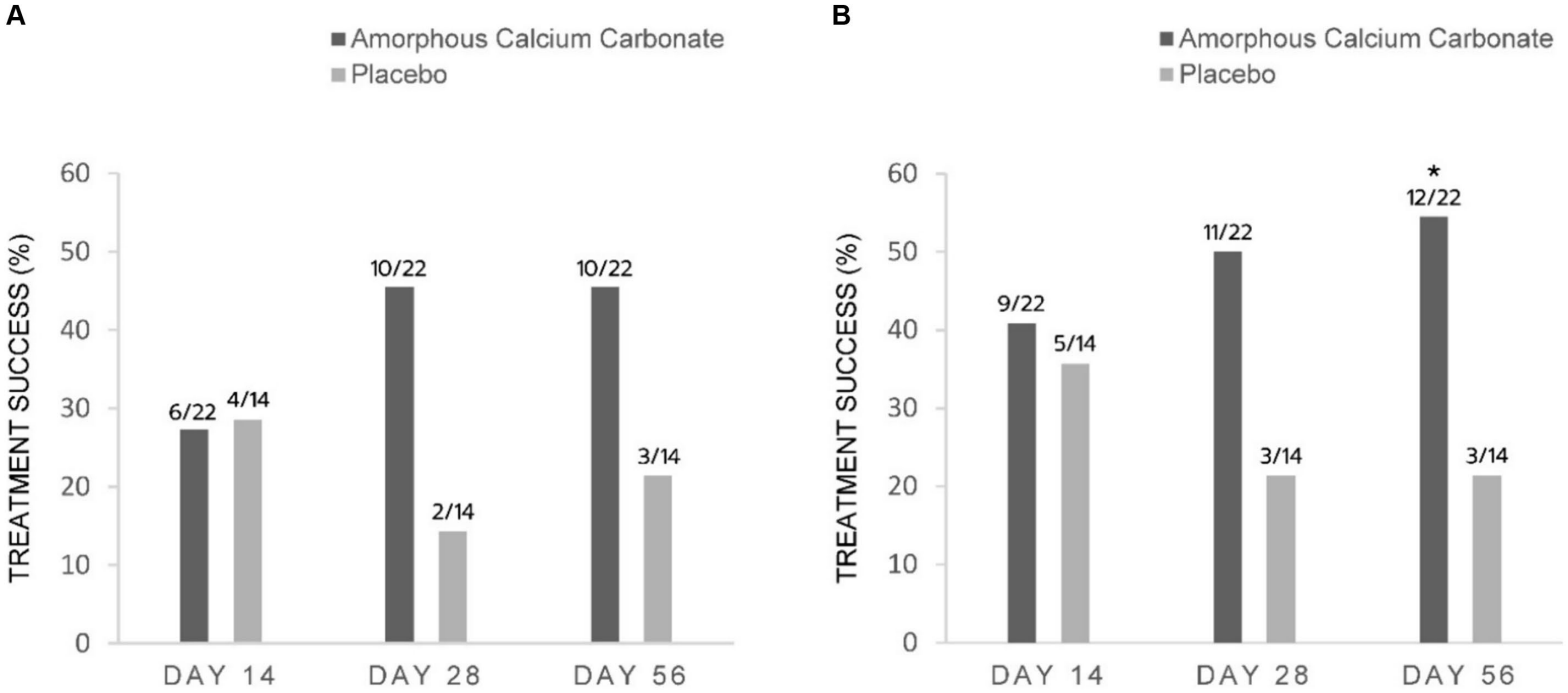
Percentage of dogs treated with either Amorphous Calcium Carbonate (ACC, *n* = 22) or placebo (*n* = 14) classified as treatment success comparing CBPI scores on day 0 to scores on days 14, 28, and 56. **(A)** Success criteria: PSS decrease ≥1, PIS decrease ≥2 from day 0; **(B)** Success criteria: PSS decrease ≥1, PIS decrease ≥1 from day 0. The number of dogs considered treatment success from each treatment group is shown above each column (fraction of the total N). * Denotes statistical significance between treatments within days (*p* = 0.05), N-1 Chi-squared test.

Based on the main efficacy endpoint of treatment success using CBPI assessments at day 56 (PSS decrease ≥1, PIS decrease ≥2), proportionally more ACC than placebo dogs were treatment successes [(45.5% 10/22) versus 21.4% (3/14), respectively]; however, these differences were not statistically significant (*p* = 0.15). Similarly, on day 28, more ACC than placebo dogs were treatment successes [45.5% (10/22) versus 14.3% (2/14), respectively]. On day 14, the proportion of treatment successes was similar between ACC and placebo [27.3% (6/22) versus 28.6% (4/14), respectively] (Figure 2A).

Using less conservative criteria for CBPI assessments at day 56 (PSS decrease ≥ 1, PIS decrease ≥ 1), significantly more ACC than placebo dogs were treatment successes on day 56 [54.5% (12/22) versus 21.4% (3/14), respectively, a difference of 33.1%, *p* = 0.05]. On days 28 and 14, the differences of 28.6 and 5.2% between treatments were not significant (Figure 2B).

**Figure 3:**
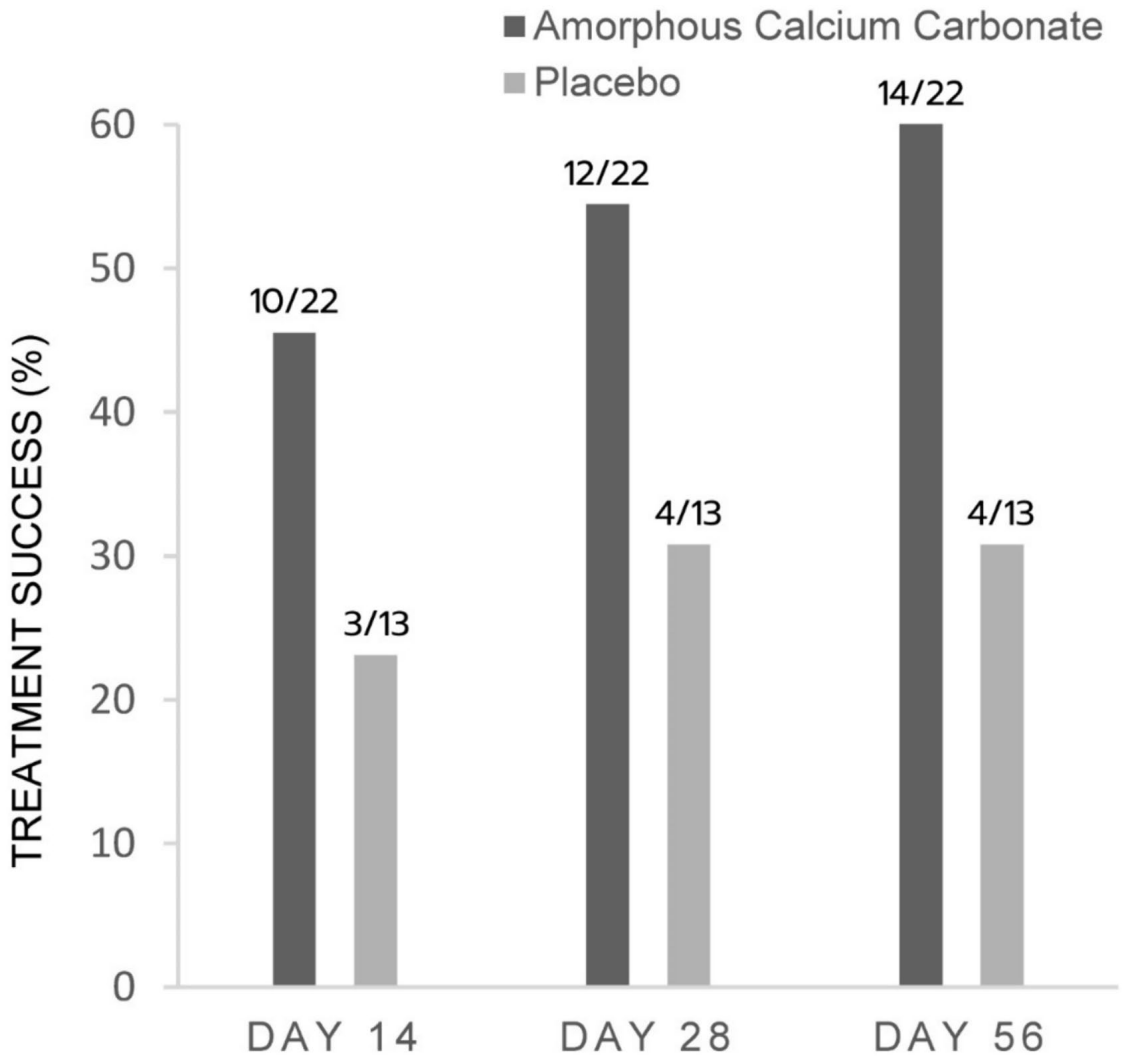
Percentage of dogs treated with either Amorphous Calcium Carbonate (ACC, *n* = 22) or placebo (*n* = 13) classified as treatment success comparing CSOM scores on day 0 to scores on days 14, 28, and 56. Success criteria: CSOM decrease ≥2 from day 0. The number of dogs considered treatment success from each treatment group is shown above each column (fraction of the total N). There were no significant differences between treatments within days (*p* > 0.05), N−1 Chi-squared test.

For CSOM assessments, proportionally more ACC than placebo dogs were treatment successes on days 56, 28, and 14, with differences of 32.8% (*p* = 0.06), 23.7, and 22.4% in treatment success, respectively. These differences were not statistically significant (Figure 3).

For efficacy assessments based on PSS, PIS, CSOM, and VOS scores, there were no significant differences between ACC and placebo treatments (*p* > 0.05) (Tables 2–5). Each of the scores was lower at day 14 than at baseline (day 0) in both treatment groups, consistent with an initially strong placebo effect. From day 14 onwards, there were significant reductions in each of the assessment scores at day 56 in the ACC-treated animals (*p* < 0.05), whereas in the placebo animals, there were no comparable changes (*p* > 0.05).

**Table 2:** Mean (±standard deviation) pain severity score (PSS) for dogs at baseline (day 0) and days 14, 28, and 56; comparisons between and within treatment groups at each time point.

	Pain severity score, PSS (mean ± SD)		
	Amorphous calcium carbonate (ACC) (*N* = 22)	Placebo (*N* = 14)	*p* value
Baseline	4.35 (±1.13)	4.63 (±1.85)	0.66
Day 14	3.56 (±1.93)^a^	3.67 (±1.92)	0.97
Day 28	3.33 (±1.68)^a^	3.79 (±2.29)	0.60
Day 56	2.75 (±1.78)^b^	3.88 (±2.13)	0.12

Repeated measures analysis of variance with baseline as a covariate.

^a, b^Within treatments, for Days 14, 28, and 56, means with different letters are significantly different (*p* = 0.0083 and 0.0054 for ACC Day 14 Vs. Day 56 and ACC Day 28 Vs. Day 56, respectively) based on a priori preplanned pairwise comparisons.

**Table 3:** Mean (±standard deviation) pain interference scores (PIS) for dogs at baseline (day 0) and days 14, 28, and 56; comparisons between and within treatment groups at each time point.

	Pain interference score, PIS (mean ± SD)		
	Amorphous calcium carbonate (ACC) (*N* = 22)	Placebo (*N* = 14)	*p* value
Baseline	5.50 (±1.47)	5.30 (±1.77)	0.78
Day 14	3.91 (±2.09)^a^	4.46 (±1.96)	0.36
Day 28	3.54 (±2.11)^a,b^	4.42 (±2.49)	0.17
Day 56	3.19 (±2.30)^b^	4.06 (±2.30)	0.18

Repeated measures analysis of variance with baseline as a covariate.

^a, b^Within treatments, for days 14, 28, and 56, means with different letters are significantly different (*p* = 0.036 for ACC Day 14 Vs. day 56) based on a priori preplanned pairwise comparisons.

**Table 4:** Mean (± standard deviation) scores for client-specific outcome measures (CSOM) for dogs at baseline (day 0) and days 14, 28, and 56; comparisons between and within treatment groups at each time point.

	Client specific outcome scores, CSOM (mean ± SD)		
	Amorphous calcium carbonate (ACC) (*N* = 22)	Placebo (*N* = 13)	*p* value
Baseline	10.0 (±1.88)	10.5 (±1.33)	0.50
Day 14	8.55 (±2.67)^a^	9.54 (±1.71)	0.45
Day 28	8.00 (±2.53)^a^	9.00 (±2.16)	0.45
Day 56	7.45 (±2.70)^b^	8.77 (±2.32)	0.23

Repeated measures analysis of variance with baseline as a covariate.

^a, b^Within treatments, for Days 14, 28, and 56, means with different letters are significantly different (*p* = 0.018 and 0.048 for ACC Day 14 vs. Day 56 and ACC Day 28 vs. Day 56, respectively) based on a priori preplanned pairwise comparisons.

**Table 5:** Mean (±standard deviation) veterinary orthopedic scores (VOS) for dogs at baseline (day 0) and days 14, 28, and 56; comparisons between and within treatment groups at each time point.

	Veterinary orthopedic scores, VOS (mean ± SD)		
	Amorphous calcium carbonate (ACC) (*N* = 22)	Placebo (*N* = 14)	*p* value
Baseline	6.54 (±2.74)	7.07 (±2.65)	0.59
Day 14	5.00 (±2.31)^a^	4.79 (±3.17)	0.83
Day 28	3.82 (±2.94)^b^	4.93 (±3.17)	0.26
Day 56	3.91 (±2.86)^b^	4.29 (±3.36)	0.71

Repeated measures analysis of variance with baseline as a covariate.

^a, b^Within treatments, for days 14, 28, and 56, means with different letters are significantly different (*p* = 0.0021 and 0.0044 for ACC Day 14 vs. 28 and ACC Day 14 vs. day 56, respectively) based on a priori preplanned pairwise comparisons.

For PSS, dogs given ACC had significantly lower scores on day 56 compared with days 14 and 28 (*p* < 0.01), whereas there were no significant differences within the placebo treatment group (*p* > 0.05).

For PIS, dogs given ACC had lower scores on days 28 and 56 compared with day 14, significantly so for day 56 (*p* = 0.04), whereas there were no significant differences within the placebo treatment group (*p* > 0.05).

For CSOM, dogs given ACC had significantly lower scores on day 56 compared with days 14 and 28 (*p* < 0.05), whereas there were no similar differences within the placebo treatment group (*p* > 0.05).

For VOS, dogs given ACC had significantly lower scores on days 28 and 56 compared with day 14 (*p* < 0.01), whereas there were no similar differences within the placebo group (*p* > 0.05).

The standardized effect sizes of ACC compared to placebo at day 56 for PSS, PIS, and CSOM were 0.58 [95% Confidence interval (CI); −1.27 to 0.09], 0.38 (95% CI; −1.05 to 0.29), and 0.52 (95% CI; −1.19 to 0.16), respectively. For within-treatment comparisons, the standardized effect sizes for the changes to day 56 for ACC in PSS and PIS scores were 1.4 and 1.57, respectively, while the effect sizes of the changes in placebo were 0.4 and 0.3, respectively. The effect sizes based on odds ratios for the proportion of dogs considered treatment success on day 56 by CBPI (PSS decrease ≥ 1, PIS decrease ≥ 2), CBPI (PSS decrease ≥ 1, PIS decrease ≥ 1), and CSOM decrease ≥ 2 were 3.1 (95% CI; 0.67 to 14.1), 4.4 (95% CI; 0.96 to 20.3), and 3.9 (95% CI; 0.91 to 17.0), respectively.

The safety population (ITT) included 41 dogs. Table 6 summarizes the type, frequency, and relation of AEs to treatments. There were no SAEs. Overall, two dogs given ACC and one dog given placebo had mild, transient, and self-limiting (maximum of 4 days) diarrhea/soft stool; these AEs could possibly have been treatment-related. Two ACC-treated dogs with a prior history of recurrent urinary tract infections (UTI) developed UTIs in the study, and these were considered possibly treatment-related. Two other possible treatment-related AEs in the ACC group were one dog with transient excessive diuresis (2 days) and one dog with transient constipation (4 days). One placebo dog was diagnosed with non-related general lymphadenopathy and was exited from the study.

**Table 6:** Adverse reactions in dogs treated with either placebo or amorphous calcium carbonate for 56 days.

	Amorphous calcium carbonate (ACC) (*N* = 26)		Placebo (*N* = 15)			
Adverse reaction	*N*	%	*N*	%	Relationship to treatment	Comment
Asthenia	1	3.8	0	0	Unrelated	Related to UTI
Cushing’s disease	1	3.8	0	0	Unrelated	Prior history
Constipation	1	3.8	0	0	Possible/probable	Duration of 4 days
Diarrhea/soft stool	2	7.7	1	6.6	Possible/probable	Duration ≤ 4 days
Diuresis excessive	1	3.8	0	0	Possible	Duration of 2 days
Traumatic ruptured muscle	1	3.8	0	0	Unrelated	
Swollen carpus	1	3.8	0	0	Unrelated	
Disk protrusion	1	3.8	0	0	Unrelated	
Urinary tract infection (UTI)	2	7.7	0	0	Possible	Prior history
General lymphadenopathy	0	0	1	6.6	Unrelated	

No treatment-related changes in clinical pathology results were identified in samples collected on day 56 (Table 7); mean and median laboratory values for days 0 and 56 were within reference ranges, and the few minor changes in clinical pathology results were not considered treatment-related. There were no differences in mean serum concentrations of total calcium and phosphorus on days 0 and 56 between treatments. Two dogs, one in each treatment group, had slightly increased total calcium concentrations (12.2 mg/dL, reference value range 7.9–12.0 mg/dL) with normal ionized calcium on day 56. These increases were not considered treatment-related as the ionized calcium is physiologically more relevant, and the changes were observed in both treatment groups.

**Table 7:** Day 0 (Baseline) and Day 56 (End of study) serum biochemistry results (Mean, Median, Min, Max) for Amorphous calcium carbonate (ACC) and placebo.

Parameter	GLU (mg/d L)	BUN/UREA (mg/dL)	CREA (mg/dL)	PHOS (mg/dL)	CA (mg/dL)	TP (g/dL)	ALB (g/dL)	GLOB (g/dL)	ALT (U/L)	ALKP (U/L)	GGT (U/L)	TBIL (mg/d L)	CHOL (mg/dL)
Reference range	70–143	7–27	0.5–1.8	2.5–6.8	7.9–12	5.2–8.2	2.2–3.9	2.5–4.5	10–125	23–212	0–11	0–0.9	110–320
**Amorphous calcium carbonate (ACC)**													
**Day 0**													
Mean	95	17	1.0	4.0	10.2	6.8	3.6	3.3	67.1	101.7	3.6	0.3	197
Median	95	16	1.1	4.1	10.4	6.5	3.5	3.2	41.0	49.0	0.0	0.3	196
(Min, max)	(75, 114)	(8, 40)	(0.3, 1.5)	(2.4, 5.6)	(8.7, 11.4)	(6.0, 10.2)	(2.8, 4.8)	(1.6, 7.5)	(10, 342)	(0.0, 479)	(0, 34)	(0.1, 0.5)	(0, 323)
**Day 56**													
Mean	96	17	1.0	4.1	10.5	6.9	3.6	3.4	65.3	112.5	5.9	0.3	213
Median	96	14.5	1.1	4.2	10.5	6.7	3.5	3.1	49.5	50.5	1.5	0.3	206
(Min, max)	(81, 109)	(6.0, 43)	(0.3, 1.5)	(2.9, 6.0)	(9.3, 12.2)	(5.9, 10.4)	(2.8, 4.9)	(2.1, 7.7)	(12, 200)	(24, 755)	(0, 61)	(0.1, 0.7)	(128, 319)
**Placebo**													
**Day 0**													
Mean	97	16	1.2	4.0	10.2	6.5	3.5	3.0	62.8	66.9	3.5	0.3	196
Median	97	15	1.2	4.1	10.3	6.6	3.2	3.2	48	57	0.5	0.3	175
(Min, max)	(86, 109)	(9, 27)	(0.9, 1.6)	(2.4, 5.4)	(9.1, 11.6)	(5.7, 7.6)	(2.8, 4.3)	(1.6, 4.2)	(29, 213)	(28, 212)	(0, 21)	(0.1, 0.3)	(141, 306)
**Day 56**													
Mean	96	20	1.2	4.4	10.6	6.8	3.6	3.2	102.9	74	0.4	0.3	223
Median	95	18	1.2	4.3	10.6	6.8	3.5	3.4	51	55	0.0	0.3	210
(Min, max)	(84, 114)	(11, 41)	(0.9, 1.6)	(3.4, 5.4)	(9.3, 12.2)	(6, 7.6)	(3, 4.3)	(2.0, 4.1)	(28, 743)	(24.0, 202)	(0.0, 2.0)	(0.1, 0.8)	(164, 306)

## 4 Discussion

This was a pilot, exploratory clinical study in dogs with spontaneous OA. The study used a randomized, placebo-controlled, double-blind design to make a preliminary evaluation of orally administered ACC compared with a placebo treatment and to gain insight into the applicability of some outcome measures to an assessment of ACC treatment and related data variability for potential utilization in future studies. As a pilot study, it was not designed to provide a robust statistical evaluation of the therapeutic efficacy of ACC because of the relatively small number of canine clinical cases used.

The results for ACC compared with placebo did not show statistically significant effects on the main efficacy outcome measures. However, contrasts between ACC and placebo for changes in PSS, PIS, CSOM, and VOS over the 8-week study do suggest possible therapeutic benefits of ACC and are supportive of conducting larger, appropriately powered studies to more robustly assess the efficacy of ACC to control pain and improve mobility in dogs with OA.

For efficacy demonstration, the Food and Drug Administration (FDA) requires sponsor companies to rely on owner assessments as the primary effectiveness measure in clinical OA drug studies in dogs, rather than on objective measurements or veterinary assessments. Moreover, the FDA requires that a predetermined definition of success is used to classify each dog as treatment success or failure and to compare the proportions of dogs that were treatment successes between the study interventions (26, 28). In this study, two validated owner questionnaires, CBPI and CSOM, were used to assess the potential efficacy of ACC. Both questionnaires have been accepted by the FDA and European Medicines Agency (EMA) for evaluating NSAIDs in the treatment of canine OA. The study confirmed the relevance of CBPI and CSOM assessments to the efficacy evaluation of ACC and provided initial efficacy and safety data as well as estimates of effect size range and data variability for the design of further studies.

In this study, predefined criteria for treatment success were used for CBPI based on a clinically relevant definition of PSS decrease ≥1 and PIS decrease ≥2 compared to day 0 and a QOL score same or better at day 56, similar to that described previously (26, 28). We also evaluated a less conservative definition of success, of PSS decrease ≥1 and PIS decrease ≥1, and a QOL score same or better at day 56, similar to that described previously (26). The observed but not statistically significant changes in CBPI and CSOM treatment success percentages for ACC are comparable to those in much larger studies of NSAIDs use in OA. In a study using CBPI and a PPP of 131 dogs/treatment and a successful definition of PSS decrease ≥1 and PIS decrease ≥2 at day 28 from day 0, grapiprant^5^ improved pain compared to placebo after 28 days (48.1 and 31.3% treatment successes, respectively) (28). Similarly, CBPI was used to evaluate the efficacy of carprofen^6^ in a 14-day OA study (*n* = 116 dogs), with success rates based on CBPI of 45.5 and 23.7% for carprofen and placebo, respectively (26). In our study, 45.5 and 54.5% of ACC-treated dogs were classified as treatment successes on day 56, compared to 21.4 and 21.4% in the placebo-treated group, using success definitions of decreases in either PSS ≥1 and PIS ≥2, or PSS ≥1 and PIS ≥ 1, respectively. The differences in success between treatments were not statistically significant based on the former definition but were when based on the latter, less conservative criteria for success. A study of 280 (ITT) OA dogs used CSOM scores and found significantly greater treatment successes after 28 days of meloxicam^7^ treatment (72.6%) than for placebo (46.9%). In the same study, differences between treatments were not observed in veterinary assessments (27). In our study treatment successes at day 56 based on CSOM scores were 63.6% in ACC-treated dogs compared with 30.8% in the placebo group; however, these differences were not significant, and treatment successes based on improvements in VOS scores were found only within the ACC group.

While no significant contrasts were found for PSS, PIS, CSOM, and VOS scores between treatment groups, it was interesting to note that significantly improved (lower) PSS, PIS, CSOM, and VOS scores were found within the ACC group on day 56, whereas no similar differences were found within the placebo group. These improvements may indicate that the effects of ACC increased over 8 weeks of the study treatment period. This should be taken into consideration in the design of future studies.

The standardized effect sizes for between-treatment comparisons on day 56 for mean PSS and PIS (0.58 and 0.38, respectively) are classified as medium (range ≥ 0.3–0.7) (30). Similarly, for treatment successes based on CBPI and CSOM (% responders) on day 56, medium effect sizes for ACC using odds ratios were obtained (3.1–4.4) (37). The effect sizes for within-treatment comparisons of changes in PSS and PIS to day 56 for ACC were greater (1.4 and 1.57) than for placebo (0.4 and 0.3, respectively). These results are comparable to those in a study of dog OA that assessed the effect sizes based on PSS and PIS of carprofen (0.55 and 0.66), tramadol (0.55 and 1.3), and placebo (0.33 and 0.35), respectively (38). It is noteworthy for NSAIDs that improvements in PSS and PIS are often apparent within 14 days, whereas for ACC with a different mode of action, the improvements appear to be more observable after 28 to 56 days. However, the time of onset of therapeutic efficacy should be clarified in a larger study.

Two mild, transient cases of soft stool/diarrhea, one case of excessive diuresis, and one case of constipation were the few possibly treatment-related adverse events observed in the ACC group during this 8-week study. Both dogs that developed UTIs had histories before enrolment of recurrent UTIs; one of these dogs also had urinary incontinence and received treatment with phenylpropanolamine before and throughout the study. Whether these UTIs were treatment-related is not clear; nevertheless, as calcium carbonate may be associated with increased urinary pH (not evaluated) and hence potentially increased risk of infection, evaluation of the effects of ACC on urinary pH and incidence of UTI will be important in future studies. The proportion of soft stool/diarrhea was similar between ACC and placebo groups (7.7 and 6.6%, respectively). There were no SAEs or other treatment-related AEs, no treatment-related changes in clinical pathology results, including serum calcium and phosphorus levels, and the use of ACC over 56 days was safe in study dogs. In humans, there is robust accumulated safety data of ACC from pre-clinical and clinical studies and post-marketing surveys in humans using the marketed nutritional supplement (see footnote 2). These data are encouraging and supportive of the safety of ACC for chronic use in dogs. Nevertheless, additional data for assurance of safety is required, including from larger numbers of clinical cases together with data from the margin of safety studies in dogs.

The pathogenesis of OA and related pain mechanisms is complex, involving the entire osteochondral unit. Existing OA therapies primarily target pain and/or inflammation; others are designed to preserve articular cartilage. Growing evidence suggests that subchondral bone remodeling in OA joints is an important target for new disease-modifying drugs (5, 7). In early OA development, acidification of the extracellular matrix by osteoclasts is required for bone osteoclastic resorption, demineralization of the subchondral bone, and the release of proteolytic enzymes, such as matrix metalloproteinases and cathepsins, leading to joint cartilage degradation (5, 8, 21, 39–41). Decreases as small as 0.1 in extracellular pH may cause a doubling of calcium resorption from subchondral bone (41). While cartilage synthesis is optimal in pH ~7.2, the pH in OA joints may reach 6.0, causing modulation of chondrocyte function and cartilage integrity and development of synovitis (20, 40). Acidity also plays an important role in pain sensation. Acid-sensing ion channels (ASICs) are important for the excitation of nociceptors by low pH, and early-stage responses to tissue injury in osteoarthritis pain may be acid-induced (42).

It is postulated but not yet established that by increasing the levels of bicarbonate locally, ACC can attenuate the local acidity and the destructive inflammatory, painful cascade in OA joints, while increased calcium levels may be available to the resorbed subchondral bone to enhance osteoblast activity and improve bone mineralization. As subchondral bone remodeling in OA is known to progress from initially increased bone resorption to later, sclerotic, poorly mineralized bone accretion (41), a good rationale exists for therapeutic approaches that target subchondral bone resorption and/or formation to modify disease progression and outcome. A number of therapeutic strategies are being investigated in human OA patients to target the remodeling process in OA subchondral bone, for example, bisphosphonates and strontium ranelate (used also for the prevention and treatment of osteoporosis by inhibiting osteoclast activity and enhancing osteoblasts), and cathepsin K inhibitors, which may act on both cartilage and subchondral bone remodeling (5).

An important limitation of this study was the small number of dogs relative to data variability. In addition, the use of a 2:1 allocation of dogs to the treatment group may reduce study power, which could have been compensated for by increasing the study sample size. For a definitive assessment of a drug’s safety and efficacy, a much larger number of dogs is clearly required, as this study did not show statistically significant efficacy differences between ACC and placebo scores. Nevertheless, it is encouraging that despite the relatively small numbers of dogs in this study, clinically relevant statistically significant differences within the ACC group for PSS, PIS, CSOM, and VOS scores and between ACC and placebo for the less conservative criteria of CBPI success consistent with the possible efficacy of ACC were identified, particularly after 8 weeks of treatment, supporting the merit of conducting full-scale clinical studies, which should be designed to clarify the time of onset of therapeutic efficacy. While recognizing that this study was small, the results facilitate the estimation of treatment group sizes for future confirmatory studie(s), which, depending on assumptions, are likely to require 150 to 190 dogs per treatment (based on, for example, 80% power and 20% attrition of cases). Larger studies may also permit stratification and evaluation of some sub-populations of OA dogs, for example, according to age, breed, body condition, and concurrent disease. Another limitation of the study arising from its pilot nature was an assessment of only one dose of ACC, which was given twice daily. Evaluation of alternative, higher and lower dosage regimens is recommended. In an adequately powered study, it is possible, for example, that a lower dose may be effective. A once-daily regimen that would support owner compliance could also be evaluated.

The current study showed that efficacy assessments based on CBPI and CSOM are applicable to ACC evaluation; however, the duration of treatment and evaluation needs to be longer than used for NSAIDS, as exemplified by the apparently larger effect sizes observed at day 56 compared with earlier time points. The potential for combined, safe use of ACC with rapid-acting analgesics in the initial phase of ACC treatment until clinical effect would be helpful to evaluate. Future studies would benefit from not only being larger but also from the use of visualization techniques (e.g., quantitative MRI) and other disease-modifying indicators observed before and after treatment to help in staging OA progression and to assess whether ACC does ameliorate OA progression.

## 5 Conclusion

The pilot study results are encouraging and support the conduction of larger studies with greater statistical power to further assess the potential efficacy and safety of ACC and to evaluate its potential novel mode of action in dogs suffering from OA.

## Data availability statement

The original contributions presented in the study are included in the article/supplementary material, further inquiries can be directed to the corresponding author.

## Ethics statement

The animal study was approved on 20 June 2021 by the ethics committee of The Animal Testing Council of Israel (approval number 20062021). The study protocol was in full compliance with Animal Welfare legislative requirements of Israel including the requirements of the Animal Testing Council of Israel. The studies were conducted in accordance with the local legislation and institutional requirements. Written informed consent was obtained from the owners for the participation of their animals in this study.

## Author contributions

HS-R: Conceptualization, Data curation, Formal analysis, Methodology, Project administration, Resources, Supervision, Visualization, Writing – original draft, Writing – review & editing. SK: Data curation, Investigation, Writing – original draft, Writing – review & editing. TGR: Formal analysis, Methodology, Validation, Visualization, Writing – original draft, Writing – review & editing.

## Footnotes

^1^Density/Densypet, Amorphical, Ness Ziona, Israel.

^2^Amorphous calcium carbonate for treatment of acidosis, Patent US20230124095A1.

^3^MedCalc Statistical Software version 20.118, MedCalc Software, Ostend, Belgium (https://www.medcalc.org).

^4^InVivoStat, version 4.6 (2022), Cambridge, UK (https://invivostat.co.uk).

^5^Galliprant, Elanco, Greenfield, IN.

^6^Rimadyl, Zoetis, Kalamazoo, MI.

^7^Metacam, Boehringer-Ingelheim, St. Joseph, MO.
